# Errata

**Published:** 1885-09

**Authors:** 


ERRATA.—In Dr. Wooten's article, for "Bichlorate of soda," read "Biborate"; for "some surgeons," read "some surgeon"; for "ripe," read "rife"; for "desolved," read "dissolved"; and for "tortured," read "tartarized." In Dr. Osborn's article, for "exacertation," read "exacerbations." Want of space prevents our citing exact lines and position of words.

